# Identification of Small Molecule Inhibitors of PTPσ through an Integrative Virtual and Biochemical Approach

**DOI:** 10.1371/journal.pone.0050217

**Published:** 2012-11-20

**Authors:** Katie R. Martin, Pooja Narang, Yong Xu, Audra L. Kauffman, Joachim Petit, H. Eric Xu, Nathalie Meurice, Jeffrey P. MacKeigan

**Affiliations:** 1 Laboratory of Systems Biology, Van Andel Research Institute, Grand Rapids, Michigan, United States of America; 2 Mayo Clinic, Scottsdale, Arizona, United States of America; 3 Laboratory of Structural Sciences, Van Andel Research Institute, Grand Rapids, Michigan, United States of America; Griffith University, Australia

## Abstract

PTPσ is a dual-domain receptor type protein tyrosine phosphatase (PTP) with physiologically important functions which render this enzyme an attractive biological target. Specifically, loss of PTPσ has been shown to elicit a number of cellular phenotypes including enhanced nerve regeneration following spinal cord injury (SCI), chemoresistance in cultured cancer cells, and hyperactive autophagy, a process critical to cell survival and the clearance of pathological aggregates in neurodegenerative diseases. Owing to these functions, modulation of PTPσ may provide therapeutic value in a variety of contexts. Furthermore, a small molecule inhibitor would provide utility in discerning the cellular functions and substrates of PTPσ. To develop such molecules, we combined *in silico* modeling with *in vitro* phosphatase assays to identify compounds which effectively inhibit the enzymatic activity of PTPσ. Importantly, we observed that PTPσ inhibition was frequently mediated by oxidative species generated by compounds in solution, and we further optimized screening conditions to eliminate this effect. We identified a compound that inhibits PTPσ with an IC_50_ of 10 µM in a manner that is primarily oxidation-independent. This compound favorably binds the D1 active site of PTPσ *in silico,* suggesting it functions as a competitive inhibitor. This compound will serve as a scaffold structure for future studies designed to build selectivity for PTPσ over related PTPs.

## Introduction

Tyrosine phosphorylation is a critical mechanism by which cells exert control over signaling processes. Protein tyrosine kinases (PTKs) and phosphatases (PTPs) work in concert to control these signaling cascades, and alterations in the expression or activity of these enzymes hallmark many human diseases [1], [2]. While PTKs have long been the focus of extensive research and drug development efforts, the role of PTPs as critical mediators of signal transduction was initially underappreciated [3]. Consequently, the molecular characterization of these phosphatases has trailed that of PTKs, and only recently has the PTP field reached the forefront of disease based-research. As validation for phosphastases in human disease, half of PTP genes are now implicated in at least one human disease [3].

The critical role of PTPs in cell function and their role in disease etiology highlight the importance of developing phosphatase agonists and inhibitors. Unfortunately, phosphatases have historically been perceived as “undruggable” for several important reasons [4]. First, phosphatases often control multiple signaling pathways and thus, inhibition of a single enzyme may not yield a specific cellular effect. Second, signaling cascades are generally controlled by multiple phosphatases and accordingly, blocking the activity of one may not sufficiently induce the desired modulation to a signaling pathway. Finally, and most importantly, phosphatase active sites display high conservation which hinders the ability to develop catalysis-directed inhibitors with any degree of selectivity [4]. Despite these pitfalls, the emerging role of PTPs in human disease etiology has necessitated a solution. Largely through use of structure-based drug design, several PTPs now represent promising targets for disease treatment. Most notably, bidentate inhibitors of PTP1B, implicated in type II diabetes and obesity, have been developed which span both the catalytic pocket and a second substrate binding pocket discovered adjacent to the active site [5], [6], [7].

Drug development around PTP1B has provided a proof-of-concept for investigations focused on additional PTP targets. Several studies have uncovered physiologically important and disease relevant functions for the classic receptor type PTP, PTPσ (encoded by the *PTPRS* gene), which underscore its potential as a biological target. PTPσ is highly expressed in neuronal tissue where it regulates axon guidance and neurite outgrowth [8], [9], [10], [11], [12]. Furthermore, it was recently reported that loss of PTPσ facilitates nerve regeneration following spinal cord injury (SCI), owing to the interaction of its ectodomain with chondroitin sulfate proteoglycans (CSPGs) [13], [14]. In addition to its neural function, PTPσ has been implicated in chemoresistance of cancer cells. First, we discovered that RNAi-mediated knockdown of PTPσ in cultured cancer cells confers resistance to several chemotherapeutics [15]. Additionally, we have discovered that loss of PTPσ hyperactivates autophagy, a cellular recycling program that may contribute to chemoresistance of cancer cells [16]. Taken together, it is apparent that modulation of PTPσ may have therapeutic potential in a range of contexts. Notably, inhibition of PTPσ could potentially provide benefit following SCI through enhanced neural regeneration. In addition, it is possible that PTPσ inhibition may yield therapeutic value in diseases in which increasing autophagy represents a promising treatment strategy (i.e., neurodegenerative diseases). Furthermore, a small molecule would provide value as a molecular probe or tool compound to interrogate the cellular functions and disease implications of PTPσ.

**Figure 1:**
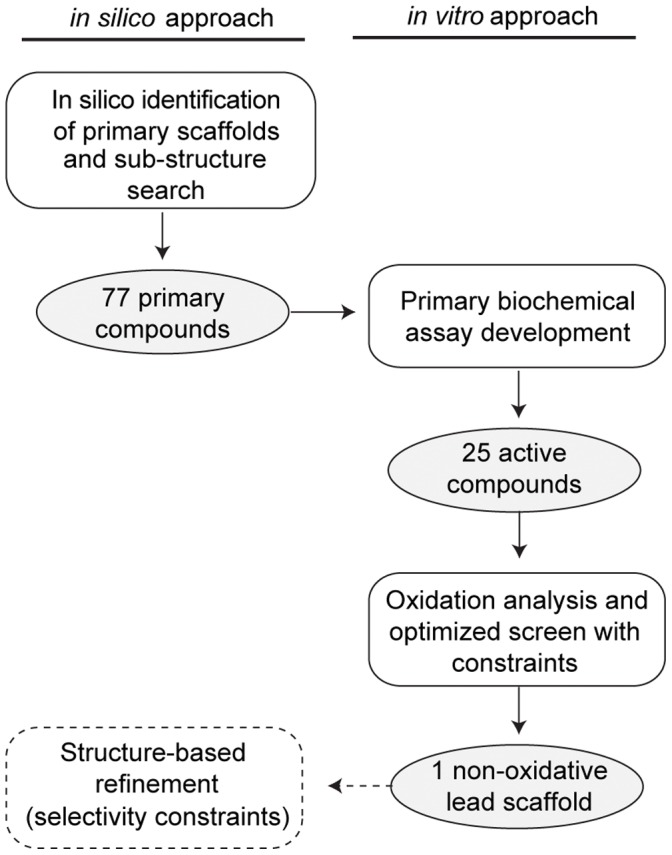
Workflow overview for PTPσ inhibitor development. *In silico* docking was performed using the crystal structure of the D1 active site of PTPσ as a target (PDB ID: 2fh7). Three structurally distinct scaffolds were chosen, along with 74 additional compounds identified by a sub-structure search of compounds in the ChemBridge, were tested *in vitro* to for the ability to inhibit PTPσ phosphatase activity. To eliminate oxidation-mediated inhibition, we optimized the biochemical assay and discovered one lead compound whose inhibition was not mediated by oxidation. Future efforts (dashed boxes and lines) will introduce selectivity into this lead compound through a detailed structural analysis of PTPσ and related PTPs.

Several approaches exist for the identification of small molecule inhibitors of phosphatases. While high-throughput screening (HTS) of compounds *in vitro* has been successfully utilized to discover inhibitors of LAR (PTPRF), PTP1B, SHP2, CD45, and others [17], the technical and physical investment is considerable as is the potential for experimental artifacts leading to false negatives and positives [17]. Alternatively, a primary screen for inhibitor scaffolds can be guided by *in silico* virtual screening. This method involves high-throughput computational docking of small molecules into the crystal structure of a phosphatase active site and selecting the molecules which bind favorably, akin to a natural substrate [18]. Following the selection of the best-scoring scaffolds, each scaffold can then be tested and validated for phosphatase inhibition *in vitro.* This approach has gained popularity as the number of enzymes with solved crystal structures has increased and it is advantageous in many ways. First, utilization of the phosphatase structure allows for the exclusion of molecules which have little chance of interacting with the active site, greatly reducing the number of scaffolds to be biochemically screened and improving the screen results. Second, an understanding of the unique structural features and residues comprising the active site as well as proximal folds or binding pockets can guide the selection and refinement of an inhibitor. Furthermore, an *in silico* approach is incredibly efficient in that it allows tens of thousands to millions of compounds to be screened virtually in a matter of weeks.

**Figure 2:**
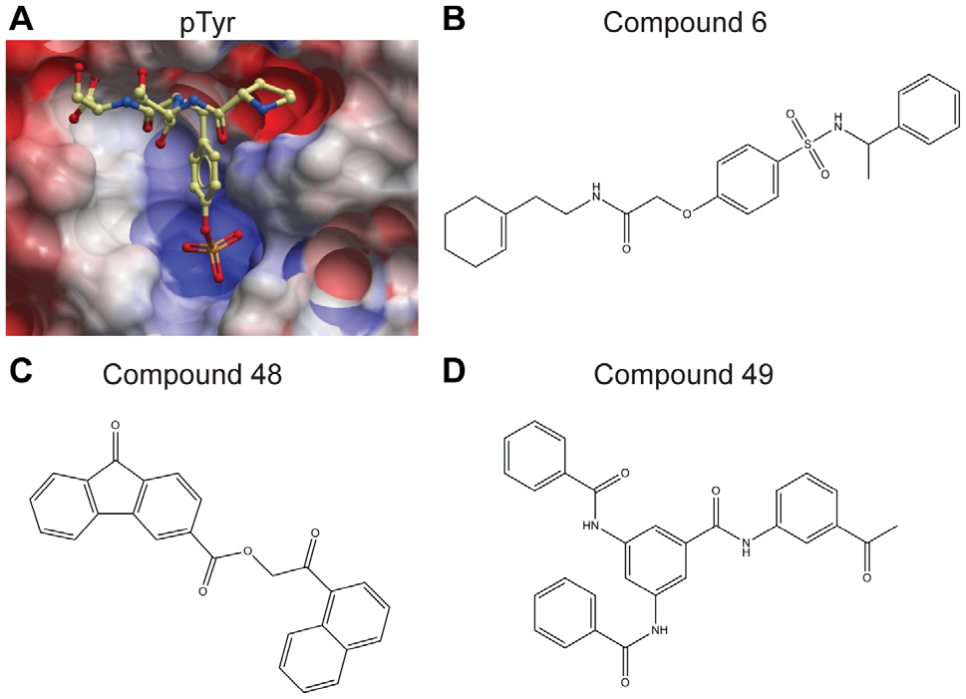
*In silico* docking identifies compounds which dock into the D1 active site of PTPσ. **(A)** The D1 domain of PTPσ docked a phosphotyrosine (p-Tyr) substrate into the active site. Surface resonance of the active site is displayed with negatively (red) and positively (blue) charged residues shown and substrate drawn in ball-and-stick form. Structures were generated with ICM software (MolSoft). **(B-D)** *In* silico screening identified structurally distinct scaffolds (compounds 6, 48, and 49), which molecularly dock into the active site, similar to the pTyr peptide. These compounds were chosen as a platform for subsequent studies based on their structural diversity and ability to inhibit PTPσ activity by at least 70% in a pilot phosphatase assay (at 10 µM), comparable to the pan PTP inhibitor, sodium orthovanadate (data not shown). Chemical structures created in ChemDraw. Compound 6: N-[2-(1-cyclohexen-1-yl)ethyl]-2-(4-{[(1-phenylethyl)amino]sulfonyl}phenoxy)acetamide. Compound 48: 2-(1-naphthyl)-2-oxoethyl 9-oxo-9H-fluorene-3-carboxylate. Compound 49: N-(3-acetylphenyl)-3,5-bis(benzoylamino)benzamide.

**Figure 3:**
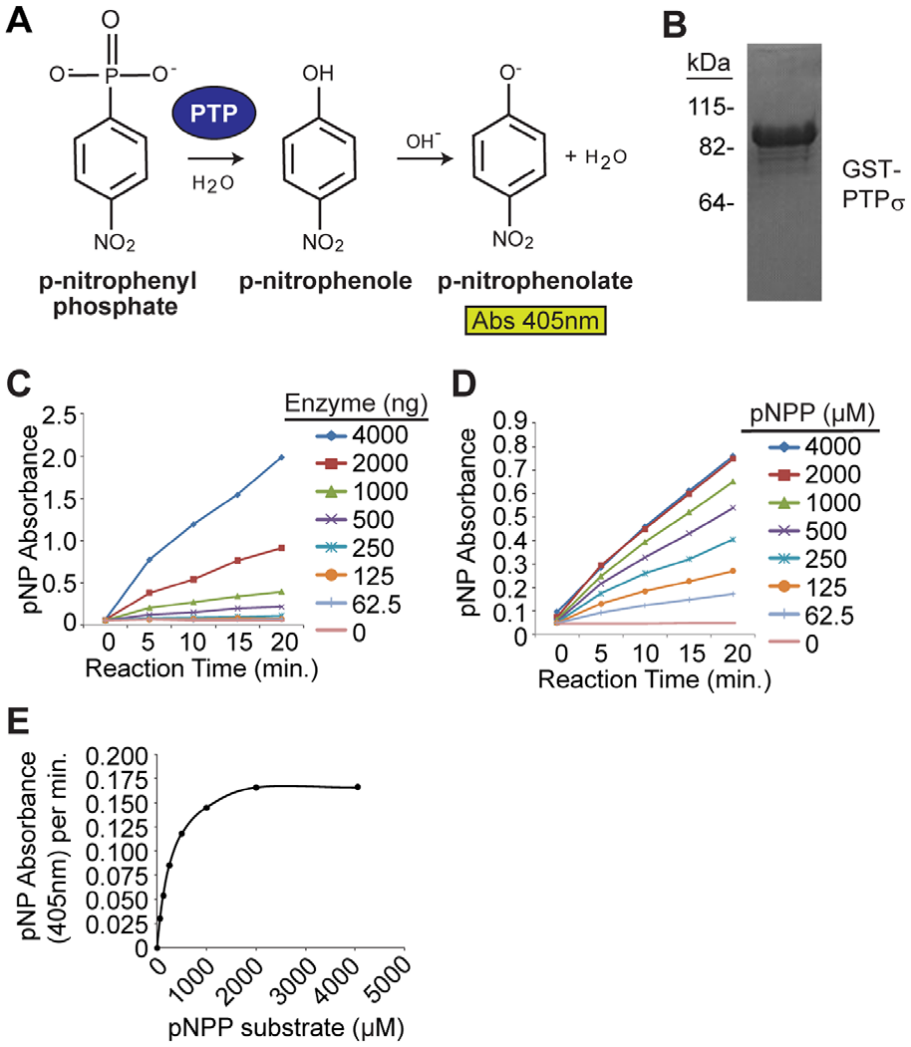
Optimization of biochemical screening conditions for PTPσ inhibition. (**A**) *Para*-nitrophenyl phosphate (pNPP) is a generic phosphatase substrate whose dephosphorylated product, para-nitrophenol (pNP), yields an intense yellow color under alkaline conditions measurable at 405 nm absorbance on a spectrophotometer. (**B**) 20 µg purified recombinant GST-PTPσ-CTF (C-terminal fragment containing the active sites) protein was resolved by SDS-PAGE and stained with coomassie blue to demonstrate purity. (**C**) The linear formation of product by various quantities of recombinant GST- PTPσ was observed through time-course reactions. pNPP-phosphatase assays were completed with a saturating dose of 1 mM pNPP. Background-corrected absorbance of dephosphorylated product are plotted by time of reaction. Each plot stems from the quantities of PTPσ indicated in the legend. (**D**) 2 µg enzyme was chosen from (A) for analysis of activity with varying doses of pNPP substrate. Each plot represents a unique dose of pNPP (indicated in the legend). Background-corrected absorbance of dephosphorylated product are plotted by time of reaction. (**E**) Initial velocities of PTPσ phosphatase activity (Y-axis; in pNP product formed per minute) were derived from the slopes of the plots in (D) at each of the indicated pNPP substrate concentrations (X-axis). From this, a Km of 250 µM is observed (denoted by dashed line).

**Figure 4:**
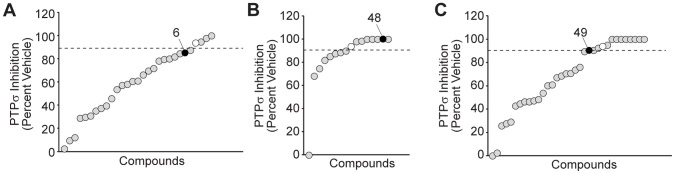
*In vitro* screen identifies active compounds which inhibit PTPσ. (**A–C**) The three *in silico*-identified scaffolds and 74 additional compounds identified by a sub-structure search of ChemBridge compounds for structural features relating to these scaffolds actives (A- similar to 6; B- similar to 48; C- similar to 49) were tested *in vitro* for potency of PTPσ. Compounds (at a final concentration of 100 µM) were pre-incubated with PTPσ for 30 minutes, then pNPP substrate added to a concentration of 200 µM and reactions continued for 30 minutes at 37°C. Dephosphorylated product was measured by its specific absorbance at 405 nm as a readout for PTPσ activity. Inhibition of PTPσ, expressed as a percent (normalized to vehicle, DMSO) is plotted for each compound. Sodium orthovanadate (Na_3_VO_4_) is a pan inhibitor of PTPs and was included as a positive control (white circles). Original scaffolds are indicated with black circles. Dashed lines denote a 90% inhibition threshold.

**Figure 5:**
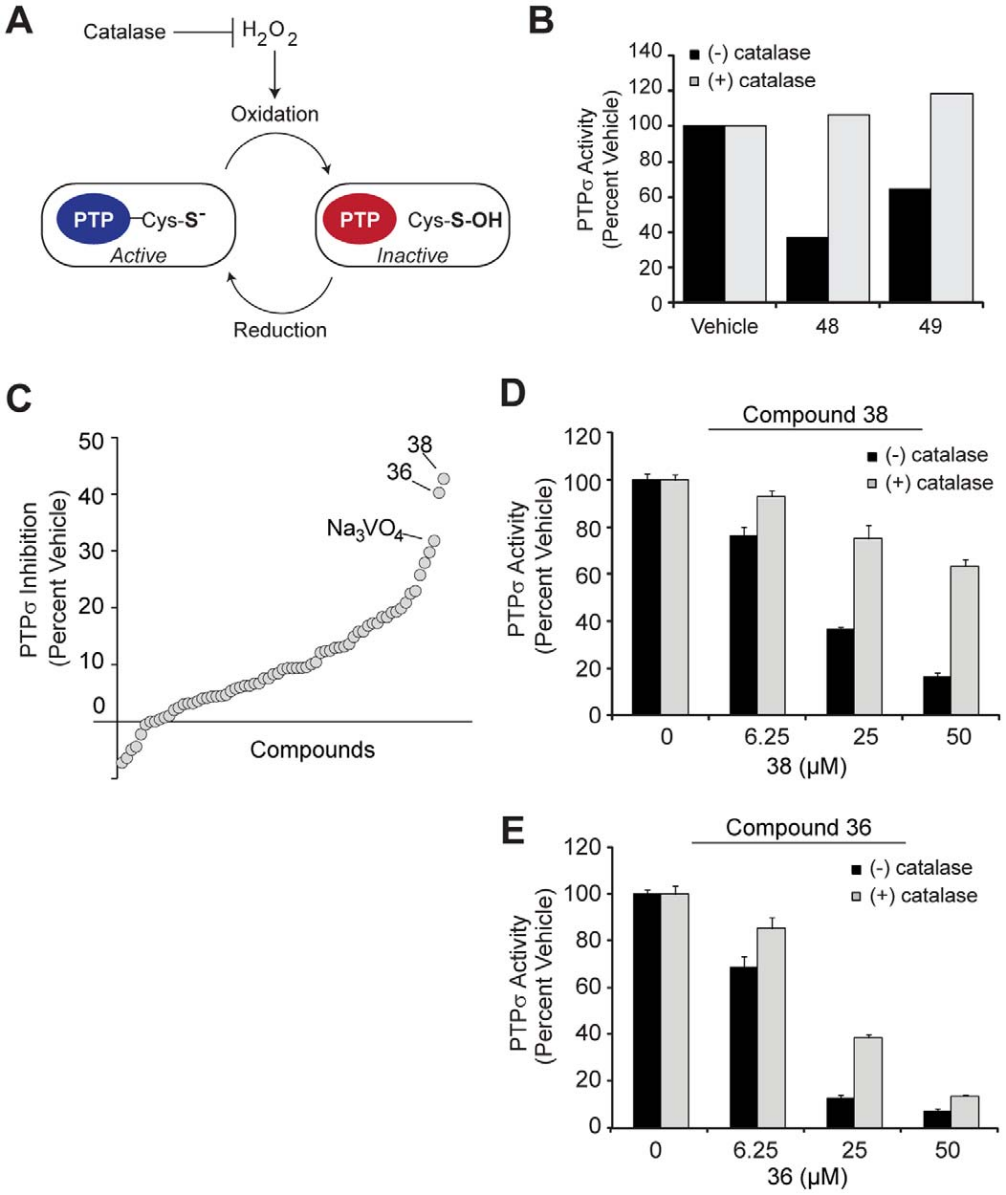
Refined biochemical screen with oxidation constraints identifies a non-oxidative molecule. (**A**) Catalase quenches hydrogen peroxide (H_2_O_2_), an oxidative species which inhibits PTP active sites through oxidation of the active site cysteine. (**B**) PTPσ activity towards pNPP was measured in the presence of DMSO vehicle, compound 48, or compound 49 as described previously. Bovine liver catalase (50 units per reaction) was included (+, gray bars) to degrade hydrogen peroxide, or excluded (−, black bars). Relative PTPσ activity was plotted (percent of activity in DMSO with or without catalase). (**C**) Sixty-six scaffolds identified *in silico* were evaluated using conditions optimized to minimize the effects of oxidation (H_2_O_2_). Compounds (10 µM) were pre-incubated with PTPσ for 10 min at 37°C then 225 µM substrate added for 15 minutes. PTPσ inhibition is plotted (normalized to vehicle, DMSO) and compounds are displayed by rank (increasing inhibition left to right). Most potent inhibitors, 36 and 38, are highlighted, as is the PTP inhibitor, Na_3_VO_4_, for reference. (**D–E**) PTPσ inhibition was measured as in (C) with compounds 38 (B) and 36 (C) with (+, gray bars) or without (−, black bars) the addition of catalase to quench hydrogen peroxide. Error bars represent standard deviation.

**Figure 6:**
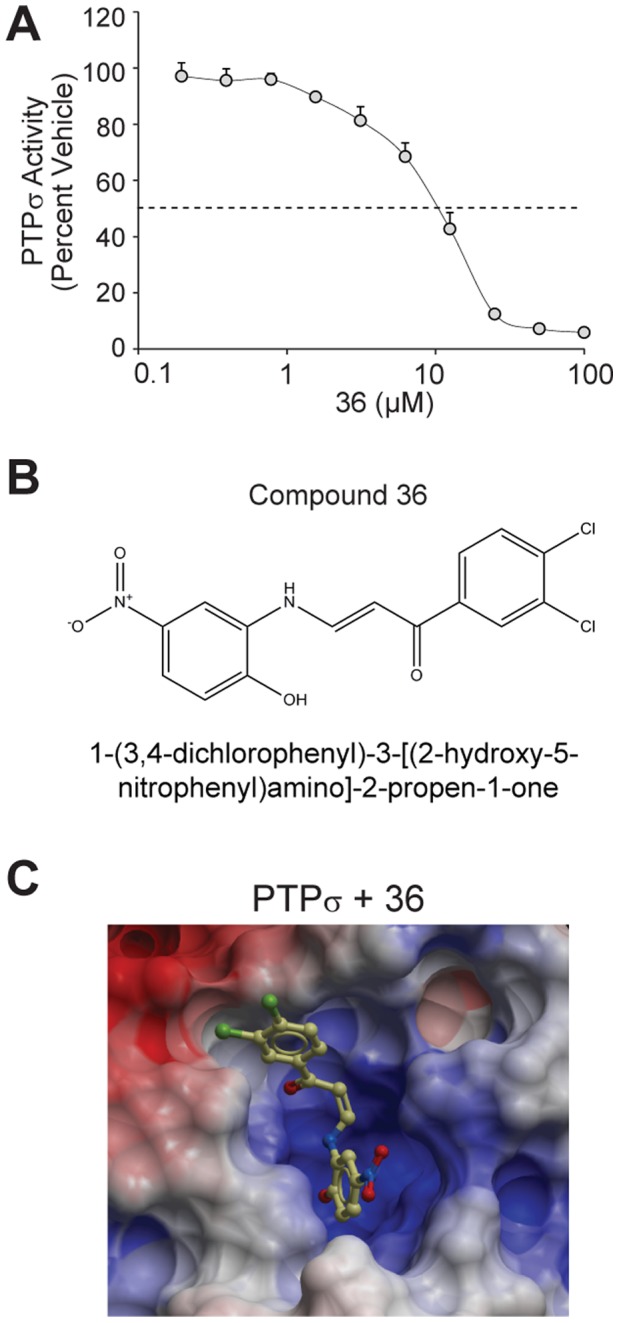
Compound 36 is a µM inhibitor of PTPσ and binds the PTPσ active site *in silico.* (**A**) Compound 36 was tested for inhibition of PTPσ. Compound 36 (at a final concentration from 0 to 100 µM) was pre-incubated with PTPσ for 10 min at 37°C. Reactions were then performed for 10 minutes at 37°C following the addition of 225 µM pNPP substrate. Relative PTPσ activity is plotted (percent of activity in DMSO). Dashed line indicates 50% inhibition (10 µM). Bars represent standard deviation from three experiments. (**B**) The structure of compound 36 is displayed. (**C**) Compound 36 was docked into the active site of PTPσ (PDB ID: 2FH7) using the D1 apo crystal structure. Molecular surface is colored by electrostatic potential. Red corresponds to negative potential and blue to positive potential. Docking was performed using the Schrödinger suite of software as described in *Methods* and figures generated using ICM (MolSoft).

The increasing number of PTP experimental structures resolved by X-ray crystallography has stimulated structure-guided efforts to identify small molecule PTP inhibitors. Drug discovery efforts focusing on PTPs are outlined in a comprehensive review written by Blaskovich, including detailed descriptions of the biological roles, target validation, screening tools and artifacts, and medicinal chemistry efforts, surrounding PTPs [19]. As outlined in this review, molecular modeling, structure-based design, and virtual screening efforts have primarily focused on hit generation and structure-guided optimization of hits for PTP1B [20], [21], [22], [23], [24], [25], [26], [27], [28], [29]. A more recent study by Park and coworkers used structure-based virtual screening to identify nine PTP1B inhibitors with significant potency [30]. Utilizing the growing knowledge base from known PTP1B inhibitors, Suresh *et al.* reported the generation of a chemical feature-based pharmacophore hypothesis and its use for the identification of new lead compounds [31]. Additional PTPs were also approached using *in silico* methodologies. Of particular interest was the study by Hu et al., which targeted the identification of small molecule inhibitors for bacterial *Yersinia* YopH and *Salmonella* SptP through differentiation with PTP1B [32]. Virtual screening also identified small molecule inhibitors of LMWPTP, SHP-2, and Cdc25 [33], [34], [35]. A review by He and coworkers underscores the progress made to date in identifying small molecule tools for the functional interrogation of various PTPs, assisted by the computational tools [36]. In addition to the classes listed above, *in silico* screening also supported the identification of Lyp inhibitors, as described in three studies by Yu, Wu, and Stanford [37], [38], [39]. Importantly, the review by He articulates both the challenges and opportunities for developing PTP specific inhibitors, serving as chemical probes to augment the knowledge of PTP biology, and to establish the basis needed to approach other PTPs currently underexplored.

In this study, we identified small molecule inhibitors targeting the active site of PTPσ. We screened compounds *in silico* to identify structurally distinct scaffolds predicted to have the most desirable binding energies. These PTPσ virtual hits, as well as additional compounds identified by a substructure and similarity search (77 in total), were iteratively tested for inhibition of PTPσ *in vitro* (**Figure 1**). While we discovered 25 active compounds with micromolar potency against PTPσ, we discovered compounds frequently catalyzed the production of oxidative species in the assay buffer, a common culprit for non-selective PTP inhibition. By optimizing the biochemical screen to include oxidation constraints, we identified one lead compound which inhibited PTPσ by a mechanism that was oxidation-independent. This lead hit was capable of docking into the active site, suggesting it functions as a competitive inhibitor. The results of this study will be used as the foundation of future structure-based refinement of PTPσ inhibitors.

## Results

### *In silico* Docking Identifies Small Molecules Targeting the PTPσ D1 Active Site

The tandem phosphatase domains of PTPσ have been crystallized in their apo form [40]. We retrieved this structure from the protein data bank (PDB ID: 2fh7) and verified its utility by molecularly docking a phosphotyrosine peptide (NPTpYS) into the catalytically active D1 domain (**Figure 2A**). We hypothesized that the active site could be exploited in the development of competitive inhibitors targeted to PTPσ. To this end, we used the ZINC database to virtually screen a library of compounds for their ability to dock into the D1 domain of PTPσ [41]. From the top scoring compounds which were most favorably bound by the active site, we identified three compounds (Compounds 6, 48, and 49) which represented structurally distinct scaffolds and demonstrated an ability to inhibit PTPσ activity in preliminary *in vitro* assays (**Figure 2B–D**). To expand these into a set of compounds for biochemical investigation, we performed a substructure search and retrieved 74 additional molecules similar to these three scaffolds from the ChemBridge compound library. This entire collection of molecules, along with the established pan-PTP inhibitor sodium orthovanadate, were analyzed for their ability to inhibit PTPσ phosphatase activity *in vitro*.

To measure the catalytic activity of PTPσ *in vitro*, we utilized the chromogenic phosphatase substrate, *para*-nitrophenyl phosphate (pNPP). The dephosphorylated product para-nitrophenol (pNP), yields an intense yellow color under alkaline conditions measurable at 405 nm absorbance on a spectrophotometer (**Figure** **3A**). We generated recombinant PTPσ and determined an amount (2 µg) that yielded linear pNP formation during the course of the phosphatase reaction while producing a maximal signal at least five-fold above background (**Figure 3B–C**). We then used initial velocities (in pNP absorbance per minute) measured across a series of pNPP substrate concentrations to calculate the K_m_ of PTPσ. The K_m_ of PTPσ was determined to be 250 µM (**Figure 3D–E**). When analyzing competitive inhibition, the mode of inhibition predicted for molecules binding the D1 active site, it is critical to use a substrate concentration at or below the K_m_ [42]. Accordingly, we used a pNPP substrate concentration less than 250 µM for inhibitor studies.

To profile the inhibition of PTPσ conferred by compounds, we pre-incubated recombinant PTPσ with each compound (100 µM) for 30 minutes, then initiated phosphatase reactions with the addition of pNPP for an additional 30 minutes. We identified 25 active compounds which inhibited PTPσ activity by 90% or more, a potency similar to that of sodium orthovanadate (Na_3_VO_4_) (**Figure 4A–C**). One of the scaffolds chosen *in silico*, compound 6, inhibited PTPσ to a lesser extent than the remaining *in silico* scaffolds, compounds 48 and 49. In fact, compounds chosen for structural similarities to compound 6 represented less than 15% of the active compounds. Therefore, we proceeded to follow up on compounds 48, 49, and similar structures.

#### Compounds inhibit PTPσ by non-selective oxidation

After identifying the most active compounds capable of inhibiting PTPσ *in vitro*, we explored the mechanism by which these molecules were reducing phosphatase activity. In particular, because PTP active sites are maintained in a reduced state for preservation of the nucleophilic cysteine which primes them for optimal activity, these enzymes are extremely sensitive to oxidation [43]. Oxidative species, such as hydrogen peroxide (H_2_O_2_), generated in the assay is a common culprit for decreased phosphatase activity [4]. To determine whether the reaction conditions were favoring H_2_O_2_-mediated inhibition of PTPσ, we repeated phosphatase assays in the presence or absence of catalase, an enzyme which converts H_2_O_2_ into water and oxygen (**Figure** **5A**). We found that catalase negated all inhibition conferred by compounds 48 and 49 (**Figure 5B**).

#### Refined screen identifies hit with minimal oxidative effect

To identify compounds with minimal oxidative effects that may better represent true competitive inhibitors, we revisited small molecules predicted to bind the PTPσ active site *in silico.* We retrieved 63 additional molecules, representing diverse structures among the top 200 scoring compounds, and tested them under screening conditions optimized to significantly diminish the potential for H_2_O_2_ generation. To achieve these conditions, we used a low dose of compound (10 µM) and reduced the pre-incubation period to only 10 minutes, as H_2_O_2_ generation and inhibition is time-dependent [4]. Under these conditions, we discovered that two compounds, 36 and 38, inhibited PTPσ by 40%, slightly more so than the equivalent dose of Na_3_VO_4_ (**Figure 5C**). We next assessed whether the inhibition mediated by these compounds involved H_2_O_2_ by determining dose-dependent inhibition in the presence and absence of catalase. Compound 38 conferred less than 50% inhibition of PTPσ at the maximal dose tested when incubated with catalase, suggesting a substantial oxidative effect (**Figure 5D**). Conversely, catalase had a less substantial effect on compound 36-mediated inhibition of PTPσ and in fact, could not prevent the inhibition conferred by relatively high doses of compounds (**Figure 5E**). This suggests that while H_2_O_2_ was partially contributing to PTPσ inhibition by compound 36, its effect was largely independent of oxidation. We used a dose-response of PTPσ inhibition to calculate the IC_50_ of compound 36 to be 10 µM (**Figure 6A**). To confirm that this molecule is capable of binding the active site of PTPσ, we molecularly docked compound 36 into the open conformation of the PTPσ D1 active site (**Figure 6B–C**). Importantly, the tyrosine-like moiety of compound 36 binds in the domain of PTPσ anticipated to bind the phosphotyrosine side chain of the known substrate.

## Discussion

Taken together, this integrative approach of computational and biochemical methods led to the identification of several small molecule inhibitors of PTPσ. *In silico* docking demonstrated that these compounds were molecularly accommodated by the D1 active site of PTPσ, similar to a natural phosphotyrosine substrate, suggesting they function as competitive inhibitors. We confirmed that one potential active site lead molecule, compound 36 [1-(3,4-dichlorophenyl)-3-[(2-hydroxy-5-nitrophenyl)amino]-2-propen-1-one], inhibits PTPσ in a dose-dependent manner with an IC_50_ of 10 µM.

Oxidation and inhibition of PTP active sites by H_2_O_2_ has been well established as a physiological mode of regulation [44]. A number of compounds, in particular those containing quinones, have been documented to inhibit phosphatases through the generation of H_2_O_2_ species [4], [45], [46]. Although the precise mechanism was not characterized, the reversal of phosphatase inhibition by compounds 48 and 49 achieved by treatment with catalase provides evidence that for at least these compounds, inhibition is partially mediated through H_2_O_2_ generation. Oxidation does not discriminate selectively for the PTPσ active site and thus, is not an ideal mechanism of inhibition for a PTPσ inhibitor. To address this, we optimized assay conditions to eliminate oxidative effects and found that compound 36 was able to inhibit PTPσ by a mechanism largely independent of oxidation. This suggests that compound 36 functions as a competitive inhibitor of PTPσ and in agreement with this, it docked favorably into the D1 active site of PTPσ *in silico*.

Although compound 36 proved potent and non-oxidative, we do not anticipate that it will be a selective inhibitor of PTPσ in its current form, owing to the high degree of sequence conservation among phosphatase catalytic domains. In particular, the residues forming the active site predominantly lie within highly conserved motifs showing little sequence variability across the entire PTP family [47]. In fact, preliminary studies suggest compound 36 displays activity towards PTP1B, in addition to PTPσ (unpublished data). This underscores the importance of our future efforts to identify and create modifications to the compound 36 scaffold which will favor selective inhibition of PTPσ.

We believe a combination of *in silico* methods and carefully optimized biochemical screening (i.e., an assay that minimizes inhibition by oxidative species) represents an useful approach to develop effective PTP inhibitors. Through the *in silico* approach described here, we were able to identify active phosphatase scaffolds while bypassing a primary assay that would entail a high-throughput biochemical screening of compounds *in vitro*. Coupling this effort with biochemical assays, we prioritized compound 36 as a lead molecule. Our future studies will include structure-based refinement of this scaffold in order to develop selective inhibitors of PTPσ. In this approach, we will characterize the activity of compound 36 against related PTPs and following, use molecular docking and structural analyses of these counter-targets to identify chemical modifications that promote selectivity for PTPσ.

## Methods

### Structural Modeling and Phosphotyrosine Substrate Docking

The crystal structure of PTPσ (PDB ID: 2FH7) was retrieved from the Protein Data Bank. The initial conformations of p-Tyr peptide (NPTpYS) were extracted from the CD45-p-Tyr peptide complex structure (PDB ID: 1YGU). The ICM program was used for protein and substrate preparation (MolSoft, La Jolla, CA). Phosphotyrosine peptide was docked into the active site of PTPσ with default parameters implemented in the ICM program.

### Virtual Screening (VS)

We used the ZINC library (version 8; University of California San Francisco) of ChemBridge compounds for virtual screening with the D1 active site of PTPσ (PDB ID: 2FH7). GOLD (Version 3.2) program was used for virtual screening and ChemScore scoring function was used to rank the top 200 hits with favorable binding energies (Cambridge Crystallographic Data Centre, Cambridge). Hydrogen bond restraints were used during molecular docking process. Molecules which formed at least one potential hydrogen bond with any of the residues, S1590, A1591, V1593, G1594 or R1595, were given higher weight during the score calculation. We used ICM clustering analysis (MolSoft) to identify 66 representative compounds from unique clustering groups. A substructure and similarity search based on compounds 6, 48, and 49 was performed using the Canvas module in Schrodinger (Schrödinger, LLC, New York, NY, 2011) and the ChemBridge compound online search engine (ChemBridge, San Diego, CA) to identify 74 additional leads.

### *In vitro* Phosphatase Assays

Compounds identified *in silico* were purchased from ChemBridge and diluted to 5 or 10 mM in DMSO. GST-tagged recombinant PTPσ containing all residues C-terminal to the transmembrane domain (BC104812 cDNA; aa 883-1501) was generated in pGEXKG [48]. GST-tagged recombinant full-length PTP1B (BC018164) was generated with a 6xHIS tag in pGEXKG. Proteins were purified from BL21 Escherichia coli after isopropyl β-D-1-thiogalactopyranoside (IPTG) induction and purity was confirmed by SDS-PAGE and coomassie blue staining. Compounds were pre-incubated with recombinant enzymes in freshly prepared phosphatase buffer (50 mM sodium acetate, 25 mM Tris-HCl, 3 mM DTT, pH 6.5) for 10 to 30 minutes, as indicated in figure legends. Following, *para*-nitrophenyl phosphate (pNPP; Sigma S0942), initially diluted in assay buffer, was added to reactions for a final volume of 100 µl and reactions were carried out in a 37°C water bath for 15 to 30 minutes. Reactions were quenched with 100 µl 1N sodium hydroxide (NaOH) and 180 µl was transferred to flat-bottom clear 96-well plates. Absorbance of pNP product at 405 nm was measured on a spectrophotometer and plotted. Background absorbance values of compound-only wells were subtracted from the corresponding reactions. DMSO was included as a vehicle control. The IC_50_ value of compound 36 was calculated using the data from Figure 6A and BioDataFit (Chang Biosciences, Castro Valley, CA).

### Docking of Compound 36 into PTPσ

Compound 36 was docked to the open conformation of PTPσ (PDB ID: 2FH7) which was retrieved from the Protein Data Bank. Docking was performed using Schrödinger’s graphical user interface Maestro (Maestro, version 9.2, Schrödinger, LLC, New York, NY, 2011). The protein was first processed using protein preparation wizard, which assigned bond orders, and added hydrogens and missing atoms, followed by minimization. Compound 36 was prepared in LigPrep (LigPrep, version 2.5, Schrödinger, LLC, New York, NY, 2011) module of Schrödinger in the OPLS-2005 force field [49] generating possible ionization states and stereoisomers for the ligand. Docking of the ligand was performed in Glide module (Glide, version 5.7, Schrödinger, LLC, New York, NY, 2011). A receptor grid was generated, defining the binding site of PTPσ, and the prepared ligand was docked using extra precision scoring function while keeping the ligand flexible. Several poses were generated which were then minimized to optimize them further using MacroModel within the OPLS2005 force field (MacroModel, version 9.9, Schrödinger, LLC, New York, NY, 2011). For Figure 6C, complexes were exported into ICM (MolSoft) and surface representations generated.
